# Perioperative use of cefazolin ameliorates postoperative cognitive dysfunction but induces gut inflammation in mice

**DOI:** 10.1186/s12974-018-1274-6

**Published:** 2018-08-22

**Authors:** Peng Liang, Weiran Shan, Zhiyi Zuo

**Affiliations:** 1 Department of Anesthesiology, University of Virginia, Charlottesville, VA 22901 USA; 2 Department of Anesthesiology and Laboratory of Anesthesia and Critical Care Medicine, Translational Neuroscience Center, West China Hospital of Sichuan University, Chengdu, Sichuan China; 3 Department of Anesthesiology and Laboratory of RNA and Major Diseases of Brain and Heart, Sun Yat-Sen Memorial Hospital, Sun Yat-Sen University, Guangzhou, 512012 Guangdong China; 4 Department of Anesthesiology, University of Virginia Health System, 1 Hospital Drive, PO Box 800710, Charlottesville, VA 22908-0710 USA

**Keywords:** Cefazolin, Gut dysbiosis, Neuroinflammation, Postoperative cognitive dysfunction

## Abstract

**Background:**

Emerging evidence indicates that long-time use of multiple antibiotics can induce cognitive dysfunction via gut dysbiosis. Cefazolin is often used for 3 to 5 days to prevent perioperative infection. This study is to detect the impact of perioperative use of cefazolin on inflammatory responses and postoperative cognition.

**Methods:**

The anti-inflammatory effect of cefazolin was determined in mouse C8-B4 microglial cells treated with lipopolysaccharide (LPS). Interleukin (IL)-6 and IL-1β at 6 and 24 h after LPS treatment were detected. Six- to 8-week-old CD-1 mice were subjected to laparotomy. Cefazolin at 300 mg/kg was injected intraperitoneally 1 h before surgery and then once per day for 5 days after surgery. Their learning and memory were assessed by Barnes maze and fear conditioning tests which started 1 week after the surgery. The brain and colon were harvested 24 h and 6 days after surgery to determine inflammatory cytokines. The colon and its luminal contents were harvested 6 and 19 days after surgery for the determination of bacteria flora. Cefazolin concentrations in the serum and brain were measured 0.5, 1, and 2 h after cefazolin injection.

**Results:**

IL-6 and IL-1β levels were decreased by 250 μg/ml cefazolin in the LPS-stimulated C8-B4 cells. Laparotomy increased the time for mice to identify the target hole in the Barnes maze on day 1 and day 8 after training sessions and reduced context-related freezing behavior in fear conditioning test. Cefazolin attenuated these surgical effects but reduced context-related freezing behavior in mice without surgery. IL-6 in the hippocampus and cerebral cortex, IL-1β in the cerebral cortex, and IL-6 and IL-1β in the serum and colon were increased 24 h after laparotomy. Cefazolin attenuated these effects. Cefazolin treatment for 5 days in mice without surgery induced colon dysbiosis and increased IL-6 and IL-1β in the colon and IL-1β in the cerebral cortex. Colon dysbiosis disappeared in mice treated with cefazolin alone but persisted in mice with surgery and cefazolin 19 days after surgery. High cefazolin concentrations in the serum but not in the brain were detected after cefazolin injection.

**Conclusions:**

These results suggest that cefazolin has a direct anti-inflammatory effect and can attenuate surgery-induced postoperative memory and learning impairment in mice. Cefazolin alone may induce cognitive dysfunction possibly by transient gut dysbiosis in mice without surgery.

## Background

Postoperative cognitive dysfunction (POCD), a severe complication in elderly surgical population [1–3], is associated with long-term disability, high social transfer costs, and increased mortality [4]. However, its mechanisms are not fully understood. Current studies on POCD mainly focus on identifying risk for POCD, contribution of surgery and/or anesthesia to the development of POCD, and possible mechanisms for surgery and anesthesia to induce POCD [2, 5, 6]. The effects of perioperative antibiotic use on postoperative cognition are not known.

Perioperative administration of antibiotics for reducing surgical infection is a consensus practice in clinical settings. Consensus guidelines most often recommend cefazolin and other cephalosporins for surgical antimicrobial prophylaxis [7]. Antibiotics are usually started 15 to 60 min before surgical incision and continued for 24 h. Empirical prolonged use of antibiotics is considered in the cases of serious surgical site infections [8]. Apart from targeting bacterial pathogens, antibiotics often indiscreetly harm some commensal gastrointestinal microbiota, allowing other pathogens and/or opportunistic bacteria to propagate, especially if antibiotics are used for a long time [9].

The potential contribution of bidirectional communication between the gut and brain to human health has been shown by association between gastrointestinal and mood disorders [10, 11]. Emerging evidence indicates that the microbiota-gut-brain axis plays an important role in the cognitive function [12, 13]. Depletion of the gut microbial community (dysbiosis) by the combination of many antibiotics for a long time can impair the cognitive function of animals [14]. However, the effects of clinically relevant use of antibiotics on cognition have not been reported.

Some antibiotics, such as minocycline, have anti-inflammatory effects [15]. Neuroinflammation is considered as a critical pathological process for POCD [5, 16, 17]. However, the effects of commonly used cefazolin on inflammation and POCD are not known.

Based on the above information, we hypothesize that cefazolin has anti-inflammatory effect and can attenuate POCD but may induce cognitive changes due to the disruption of gut microbiota. To address these hypotheses, in vitro and in vivo experiments were performed.

## Methods

### Cell culture and treatment

C8-B4 cells (CRL-2540TM, American Type Culture Collection, Manassas, VA, USA), a mouse microglial cell line [18], were grown in Dulbecco’s modified Eagle medium with 10% fetal bovine serum. Cells were maintained at 37 °C in a humidified atmosphere with 5% CO_2_. Cells were plated at 5 × 10^5^ cells/well in 6-well plates and treated with various cefazolin concentrations [50, 100, or 250 μg/ml, diluted by normal saline (NS)] for 6 or 24 h. Control cells were treated with medium and NS. For lipopolysaccharide (LPS) treatment, culture medium was replaced with fresh medium containing 1 μg/ml LPS. This incubation was for 6 h or 24 h. Culture medium was harvested to measure interleukin-1β (IL-1β) and IL-6 by using ELISA kits (MLB00C, M6000B, R&D System, Minneapolis, MN, USA).

### Animals and experimental groups

Six- to 8-week-old CD-1 male mice (weighing 31–36 g) were used. The mice were housed in cages (5 mice/cage) on a 12-h light/dark cycle with free access to water and food. For assessment of mouse behavior, observers were blinded to the group assignment and treatments of mice.

Five separate cohorts of mice were used in the experiments. In the first cohort, mice were randomized by a SPSS-generated random number assignment to one of four groups (*n* = 20 in each group): control, cefazolin, surgery, and surgery plus cefazolin. These mice were used for learning and memory tests (Barnes maze and fear conditioning tests) which started 7 days after the surgery. The second cohort mice (*n* = 5–7 in each group), also in the four groups, were used to obtain the serum, hippocampus, cerebral cortex, and colon for ELISA analyses of IL-1β and IL-6 at 24 h after surgery. The third cohort mice (*n* = 7 in each group), also in the four groups, were used for harvesting the hippocampus, cerebral cortex, and colon including its luminal contents 6 days after the surgery for ELISA analyses or 16S rRNA analyses. The fourth cohort mice (*n* = 5–6 in each group) with the same four groups were used to harvest the colon and its luminal contents 19 days after the surgery for 16S rRNA analyses. The fifth cohort mice (*n* = 7 in each group) were used to measure cefazolin concentrations in the blood and brain. Mice received one dose of cefazolin and their blood and brain were harvested 0.5, 1, or 2 h after the injection of cefazolin for measuring cefazolin by high-performance liquid chromatography (HPLC).

### Exploratory laparotomy model

All mice were anesthetized by 1.5% isoflurane for exploratory laparotomy as we did before [19]. Briefly, mouse was kept at spontaneous respiration with an inspiration of 50% O_2_. Rectal temperature was monitored and maintained at 37 °C with the aid of a heating blanket (TCAT-2LV, Physitemp instruments Inc., Clifton, NJ, USA). The hair in the abdomen was shaved and the abdominal area was sterilized with iodine. Bupivacaine (0.25%, 3 mg/kg) was infiltrated to the incision site before surgery. The abdominal cavity was opened by an incision from the xiphoid process to the superior margin of the pubic symphysis. The right colon, diaphragmatic surface of the liver, spleen, kidneys, and bladder were explored by a cotton tip wetted with NS to mimic clinical exploratory laparotomy. To ensure similar degree of stimulation, every mouse was explored by the same set of procedure. After the peritoneum and skin were closed separately, all animals received a subcutaneous injection of 3 mg/kg bupivacaine to the wound again for postoperative analgesia. The total duration of anesthesia was 2 h.

### Cefazolin treatment

Cefazolin (1 g/vial, APOTEX CORP, Weston, FL, USA) was dissolved by NS to 100 mg/ml. Cefazolin at 300–500 mg/kg in mice is used for prevention of wound infection according to a previous report [20]. In this study, 10 mg cefazolin in 0.1 ml was intraperitoneally injected 30 min before surgery and then once every day for 5 days, while 0.1 ml NS was intraperitoneally injected in the control group and surgery only group.

### Barnes maze

Seven days after surgery, Barnes maze was used to test the spatial learning and memory of mice as we did previously [21]. The Barnes maze instrument (SD Instruments, San Diego, CA, USA) is a round platform with 20 peripheral holes, one of which is equipped with a dark box. This hole is called target hole. After the mouse was placed in the middle of the platform, aversive noise (85 dB) and very bright light from a 200-W bulb were used to stimulate mice to seek and enter the target hole. Mice were trained in a spatial acquisition phase (3 min per trial, 2 trials each day for 4 days and 2-h interval between trials). The spatial memory test was conducted 1 day after training (short-term retention) and 8 days after training (long-term retention). Mice had a rest from the short-term to long-term retention test. The latency to enter the target hole in every trial was calculated using an ANY-Maze video tracking system (SD Instruments).

### Fear conditioning

Fear conditioning test was conducted the next day after the Barnes maze test [21]. A transparent plastic chamber wiped with 70% alcohol placed in a relatively dark room was used to accommodate mice encountering three tone-foot shock pairings with 1-min interval (tone, 2000 Hz/85 dB/30 s; foot shock, 0.7 mA/2 s). Mice were returned to the same chamber for 8 min without tone and shock 24 h after training. The freezing behavior was recorded in an 8-s interval. Two hours later, mice were placed in another chamber with a different context, which was wiped with 1% acetic acid and was in a relatively light room. The tone stimulus was added for three cycles (30 s per cycle, 1 min inter-cycle interval, total 4.5 min) after 3 min without any stimuli in the cage. All freezing behavior during 4.5 min with tone stimulus was recorded by a video camera and was scored by an observer who was blinded to group assignment.

### Blood, brain, and colon tissue harvesting

Mice were anesthetized with isoflurane. Thoracotomy was performed for obtaining blood from the heart. Blood was centrifuged at 1300 relative centrifugal field for 20 min at 4 °C after it had been placed at 4 °C for 2 h for serum collection. Those mice for brain tissue harvesting were perfused with NS. The bilateral hippocampi and their overlaying cerebral cortices were dissected out for ELISA assay. The acceding colon was harvested for ELISA and 16S rRNA assessment. All dissection procedures were performed on ice.

### ELISA of interleukins the in serum, brain, and colon

IL-6 and IL-1β in the serum, brain, and colon were detected by using ELISA kits (R&D SYSTEM). The tissues (hippocampus, cerebral cortex, or colon) were homogenized on ice in the RIPA buffer (25 mM Tris-HCl with pH 7.6, 150 mM NaCl, 1% sodium deoxycholate, and 0.1% SDS) (Thermo Scientific, Rockford, IL, USA) and a protease inhibitor cocktail (10 mg/ml aproteinin, 5 mg/ml peptastin, 5 mg/ml leupetin, and 1 mM phenylmethane sulfonylfluoride) (Sigma-Aldrich, St. Louis, MO, USA). After being centrifuged for 20 min (13,000*g*, 4 °C), the supernatant was collected for ELISA detection according to the manufacturer’s instruction. Cytokines in the serum were detected in the same way. The amount of IL-6 and IL-1 β in each sample except for those in the serum was normalized by its protein content.

### 16S rRNA PCR method

Colon tissues including their luminal contents were harvested. DNA was extracted with Power Lyzer Power soil DNA isolation kit (Mo Bio Laboratories, Inc., Carlsbad, CA, USA) according to the manufacturer’s instruction. Total of 50 ng DNA was used as DNA template for 16S rRNA PCR. The V3 and V4 region of 16S rRNA was amplified by the primers F: 5′-CCTACGGGAGGCTGCAG-3′ and R: 5′-GACTACACGGGTATCTAATCC3′ [22] with the following procedure: 95 °C for 3 min and 25 cycles of 95 °C for 30 s, 55 °C for 30 s, 72 °C for 30 s, and then 72 °C for 5 min, stored at 4 °C. The amplicons were applied to electrophoresis with 1% agarose gel. Products of 500 bp were quantified.

### Cefazolin concentration measurement

Cefazolin concentration in the mouse serum and brain tissues was determined by HPLC. The blood, hippocampus, and cerebral cortex were harvested 0.5, 1, or 2 h after the intraperitoneal injection of 300 mg/kg cefazolin. The procedure to harvest these tissues and the preparation of serum was the same as described in the “Blood, brain, and colon tissue harvesting” section. The brain tissues were then homogenized in five times of NS volumes. After centrifugation, the supernatant was used for HPLC. HPLC analyses of the samples were performed as described before [23] with an Agilent ZORBAX-C18 column, a mobile phase of methanol:50 mM ammonium acetate aqueous solution = 15:85 (pH was adjusted by glacial acetic acid to 4.30), and a detection wavelength at 272 nm. The mobile phase speed was 1 ml/min. After standard curve of cefazolin concentrations was established, 50 μl of samples was mixed with 50 μl of pure water, vortexed for 10 s, and then mixed with 200 μl of 6% perchloric acid solution. The samples were vortexed for 30 s and then centrifuged at 20,000 rpm for 10 min. Twenty microliters of the supernatant was used for HPLC.

### Statistical analysis

Parametric data with normal distribution are presented as mean ± S.E.M. (*n* ≥ 5). One-way or two-way repeated measures analysis of variance followed by Tukey test was used to analyze the data from the training sessions of Barnes maze test within the same group or between groups, respectively. Other data were analyzed by one-way analysis of variance followed by the Tukey test with normally distributed data or by one-way analysis of variance on ranks followed by the Tukey test with non-normally distributed data. Differences were considered statistically significant at *P* < 0.05. All statistical analyses were performed with SigmaStat (Systat Software, Inc., Point Richmond, CA, USA).

## Results

### Cefazolin had a direct anti-inflammatory effect on C8-B4 cells stimulated by LPS

**Fig. 1:**
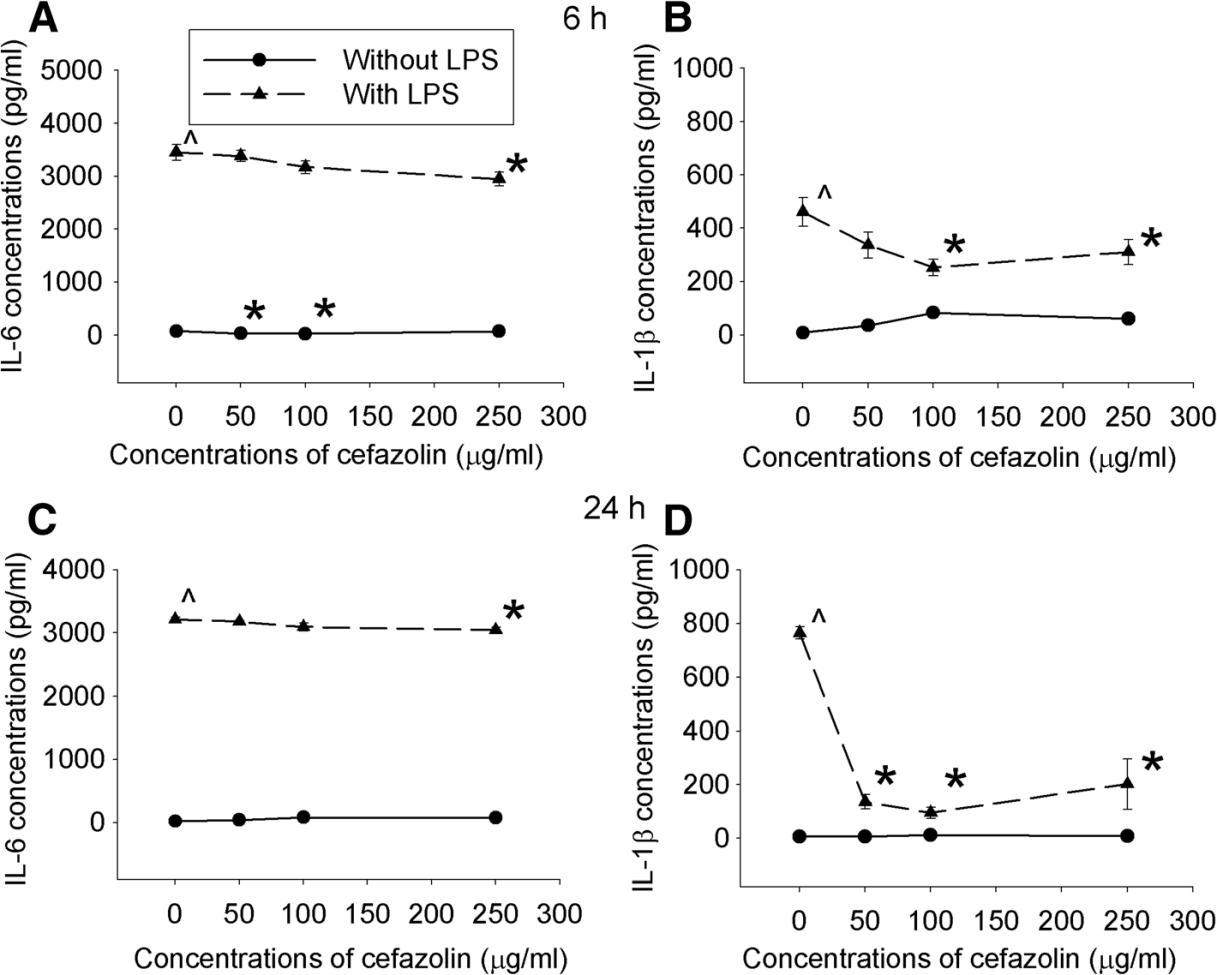
Cefazolin inhibited proinflammatory cytokine production. C8-B4 cells were incubated with or without 1 μg/ml LPS in the presence or absence of various concentrations of cefazolin. Culture medium was harvested after the cells were incubated for 6 (**a**, **b**) or 24 h (**c**, **d**). Results are mean ± S.E.M. (*n* = 7–9). *P < 0.05 compared with corresponding cells without cefazolin treatment, ^*P* < 0.05 compared with control cells

IL-6 and IL-1β levels in the culture medium of cells treated with or without different concentrations of cefazolin under control condition were very low, and cefazolin at 50 and 100 μg/ml inhibited IL-6 production (Fig. 1). LPS increased IL-6 and IL-1β production. The increase of IL-1β was inhibited by all doses of cefazolin but the increase of IL-6 was inhibited only by cefazolin at 200 μg/ml, the highest dose tested. This cefazolin effect was similar whether the culture medium was harvested after cells were incubated with LPS and cefazolin for 6 h or for 24 h (Fig. 1). These results suggest that cefazolin can inhibit LPS-induced inflammatory response.

**Fig. 2:**
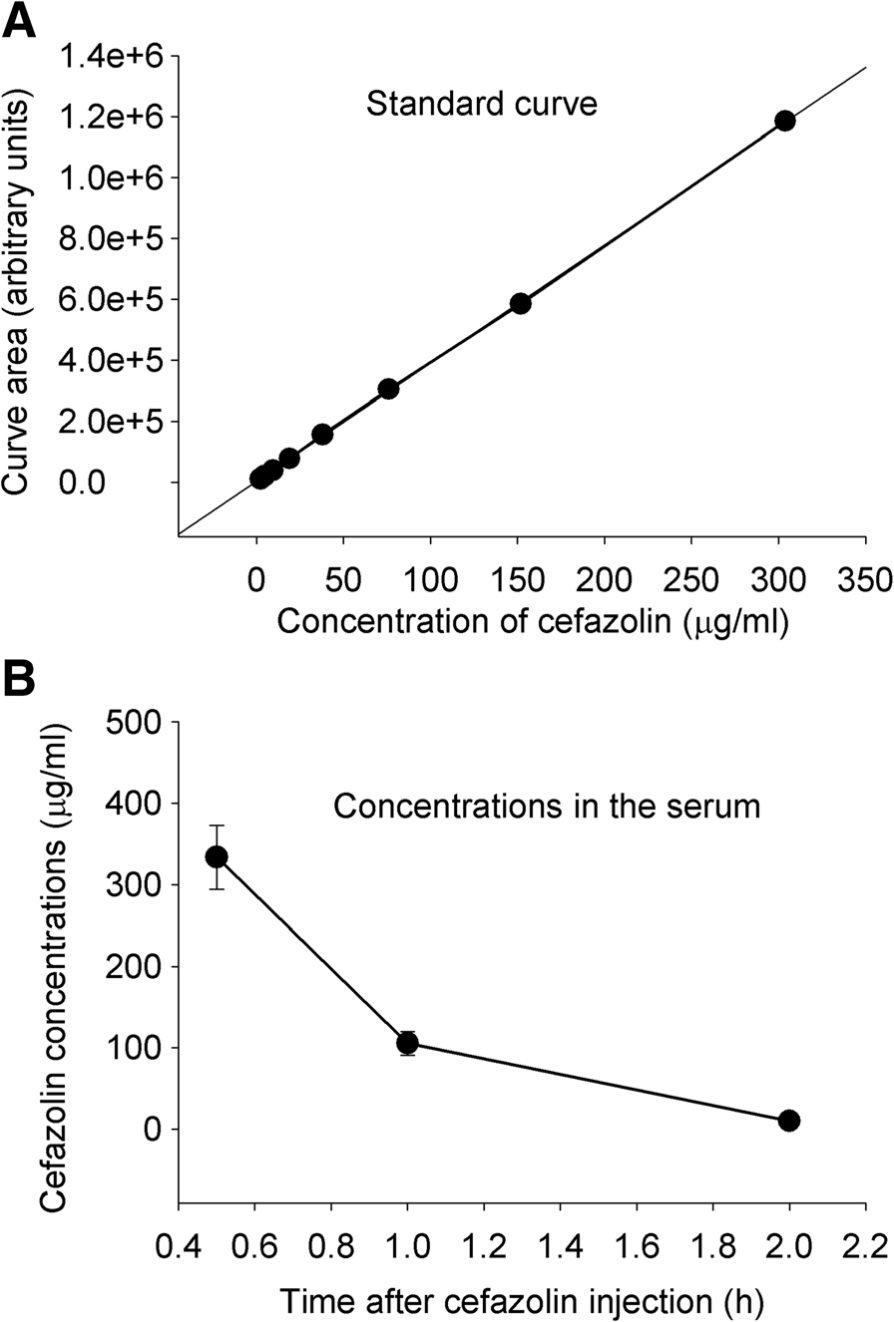
Cefazolin concentrations in the serum. Six- to 8-week-old mice received 300 mg/kg cefazolin intraperitoneally. Blood was harvested 0.5, 1, or 2 h after the injection. **a** Cefazolin concentration standard curve. **b** Cefazolin concentrations in the serum. Results are mean ± S.E.M. (*n* = 7)

The serum concentrations of cefazolin were higher than 300 μg/ml at 30 min after the cefazolin injection (Fig. 2). These concentrations were higher than those needed to inhibit LPS-induced cytokine production in microglial cultures as shown above. However, we did not detect any cefazolin in the cerebral cortex and hippocampus with a detection limit at 1.5 μg/ml by our assay (Fig. 2). These results suggest that cefazolin does not cross the blood-brain barrier, consistent with previous findings [24, 25].

### Cefazolin improved learning and memory in mice after surgery and might impair learning and memory in mice without surgery

**Fig. 3:**
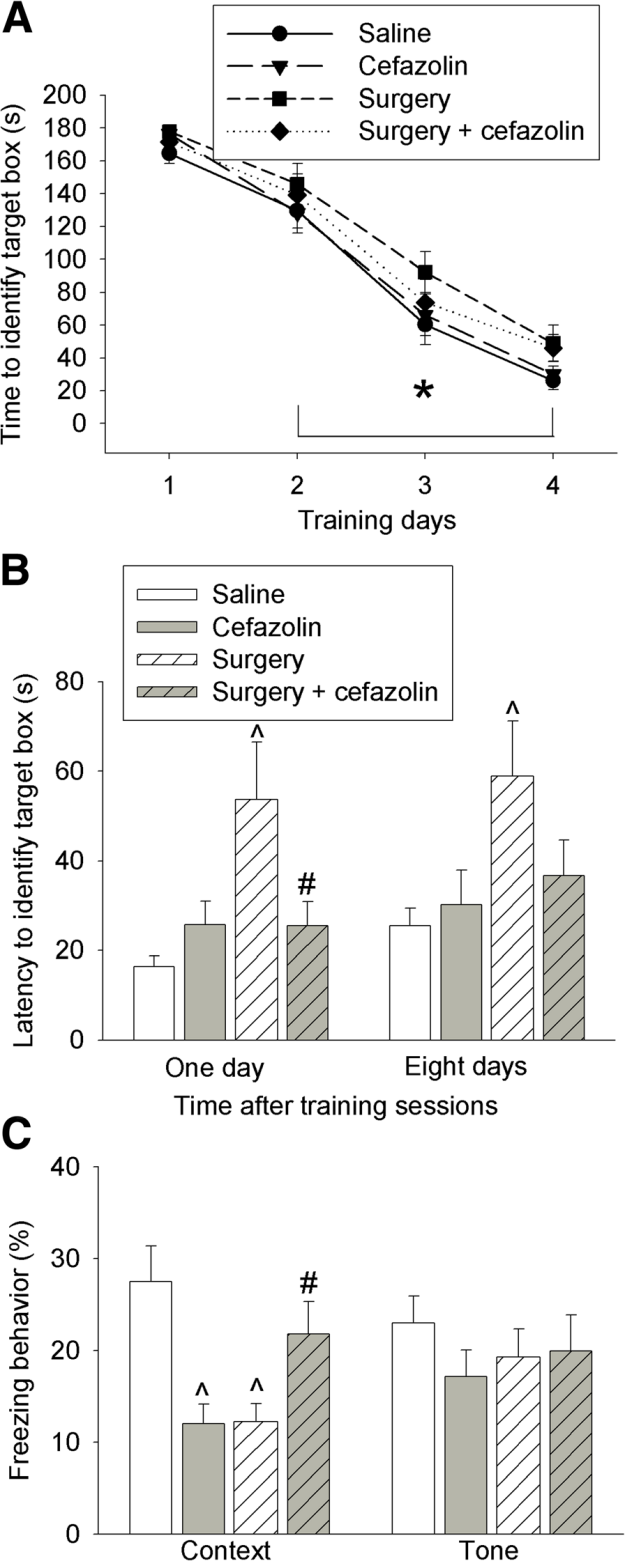
Effects of cefazolin on learning and memory. Six- to 8-week-old male CD-1 mice were subjected to laparotomy with or without cefazolin and tested in Barnes maze and fear conditioning from 1 week after the surgery. **a** Training sessions of Barnes maze. **b** Memory phases of Barnes maze. **c** Fear conditioning. Results are mean ± S.E.M. (*n* = 20). *P < 0.05 compared with the corresponding values on day 1, ^*P* < 0.05 compared with control mice, #*P* < 0.05 compared with mice with laparotomy

The time for mice to identify target box in Barnes maze test was decreased with the increase in training sessions. This time on training days 2, 3, and 4 was shorter than that on day 1 in all four groups (Fig. 3a). Surgery was a significant factor to influence the time to identify the target box during training sessions [*F*(1,38) = 5.179, *P* = 0.029]. Cefazolin was not a significant factor to influence this time no matter whether animals were with or without surgery. Surgery increased the time for mice to identify the target box 1 day or 8 days after the training sessions. Cefazolin attenuated this increase (Fig. 3b). Surgery decreased the context-related fear conditioning. This decrease was also attenuated by cefazolin (Fig. 3c). These results suggest that surgery induces learning and memory dysfunction, which is consistent with our previous findings [18, 21]. Our results also suggest that cefazolin attenuates this dysfunction.

Interestingly, cefazolin reduced context-related fear conditioning in mice without surgery (Fig. 3c). This result indicates that cefazolin may impair learning and memory of the mice under control condition.

### Effects of surgery and cefazolin on interleukin expression

**Fig. 4:**
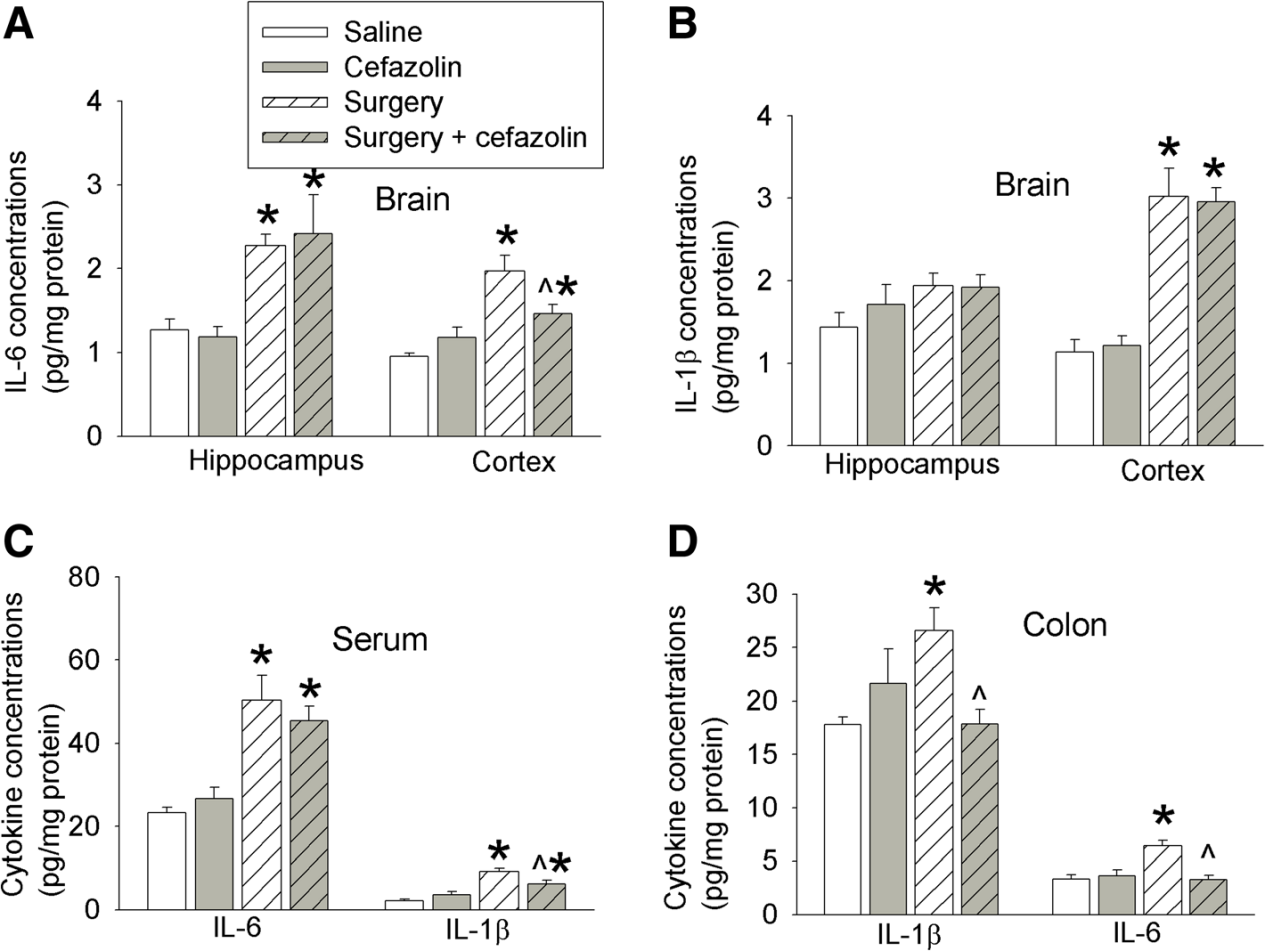
Effects of cefazolin on cytokine production at 24 h after laparotomy. Six- to 8-week-old male CD-1 mice were subjected to laparotomy with or without cefazolin. The cerebral cortex, hippocampus, blood, and colon were harvested 24 h after the surgery. **a** IL-6 in the brain. **b** IL-1β in the brain. **c** IL-6 and IL-1β in the serum. **d** IL-6 and IL-1β in the colon. Results are mean ± S.E.M. (*n* = 5–7). *P < 0.05 compared with control mice, ^*P* < 0.05 compared with mice with laparotomy

IL-6 was increased in the hippocampus and cerebral cortex 24 h after surgery. Cefazolin attenuated this increase in the cerebral cortex (Fig. 4a). Surgery also increased IL-1β in the cerebral cortex (Fig. 4b). In addition, IL-6 and IL-1β in the serum and colon were increased 24 h after the surgery. The increase of IL-1β in the serum and the increase of IL-6 and IL-1β in the colon were inhibited by cefazolin (Fig. 4c, d). These results suggest that surgery induces systemic, brain, and colon inflammatory response and that cefazolin attenuates this response 24 h after surgery.

**Fig. 5:**
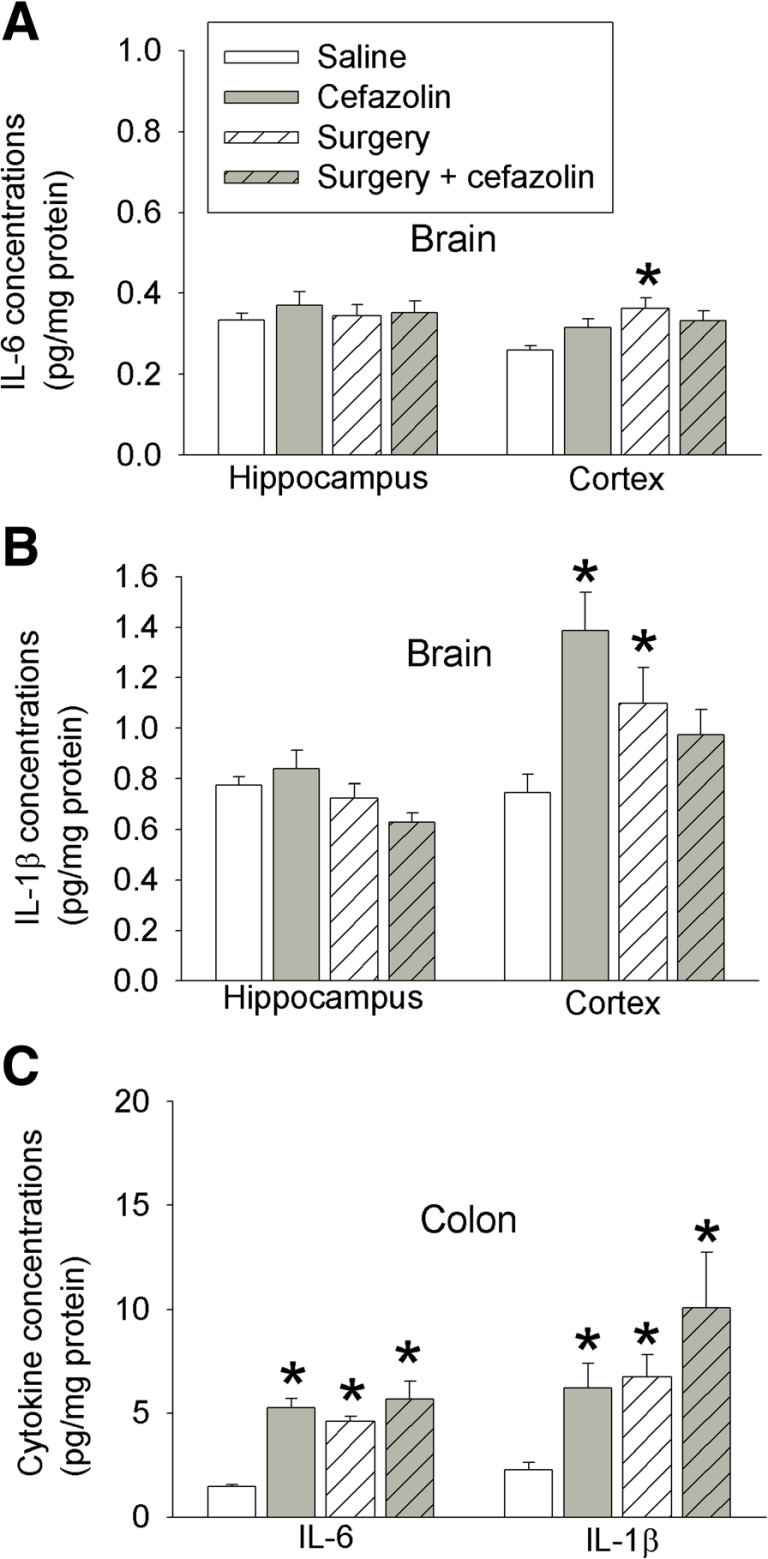
Effects of cefazolin on cytokine production at 6 days after laparotomy. Six- to 8-week-old CD-1 mice were subjected to laparotomy with or without cefazolin. The cerebral cortex, hippocampus, and colon were harvested 6 days after the surgery. **a** IL-6 in the brain. **b** IL-1β in the brain. **c** IL-6 and IL-1β in the colon. Results are mean ± S.E.M. (*n* = 7). *P < 0.05 compared with control mice, ^*P* < 0.05 compared with mice with laparotomy

IL-6 and IL-1β in the cerebral cortex were increased 6 days after surgery. This increase was not inhibited by cefazolin (Fig. 5a, b). Instead, cefazolin alone increased IL-1β in the cerebral cortex (Fig. 5b). Also, cefazolin alone and surgery alone increased IL-6 and IL-1β in the colon 6 days after the onset of cefazolin use or 6 days after the surgery (Fig. 5c). These results suggest that mice with surgery remain to have inflammatory response in the brain and colon 6 days after surgery and that cefazolin may induce inflammation in the colon and brain at a late phase.

### Cefazolin used for 5 days induced colon dysbiosis

**Fig. 6:**
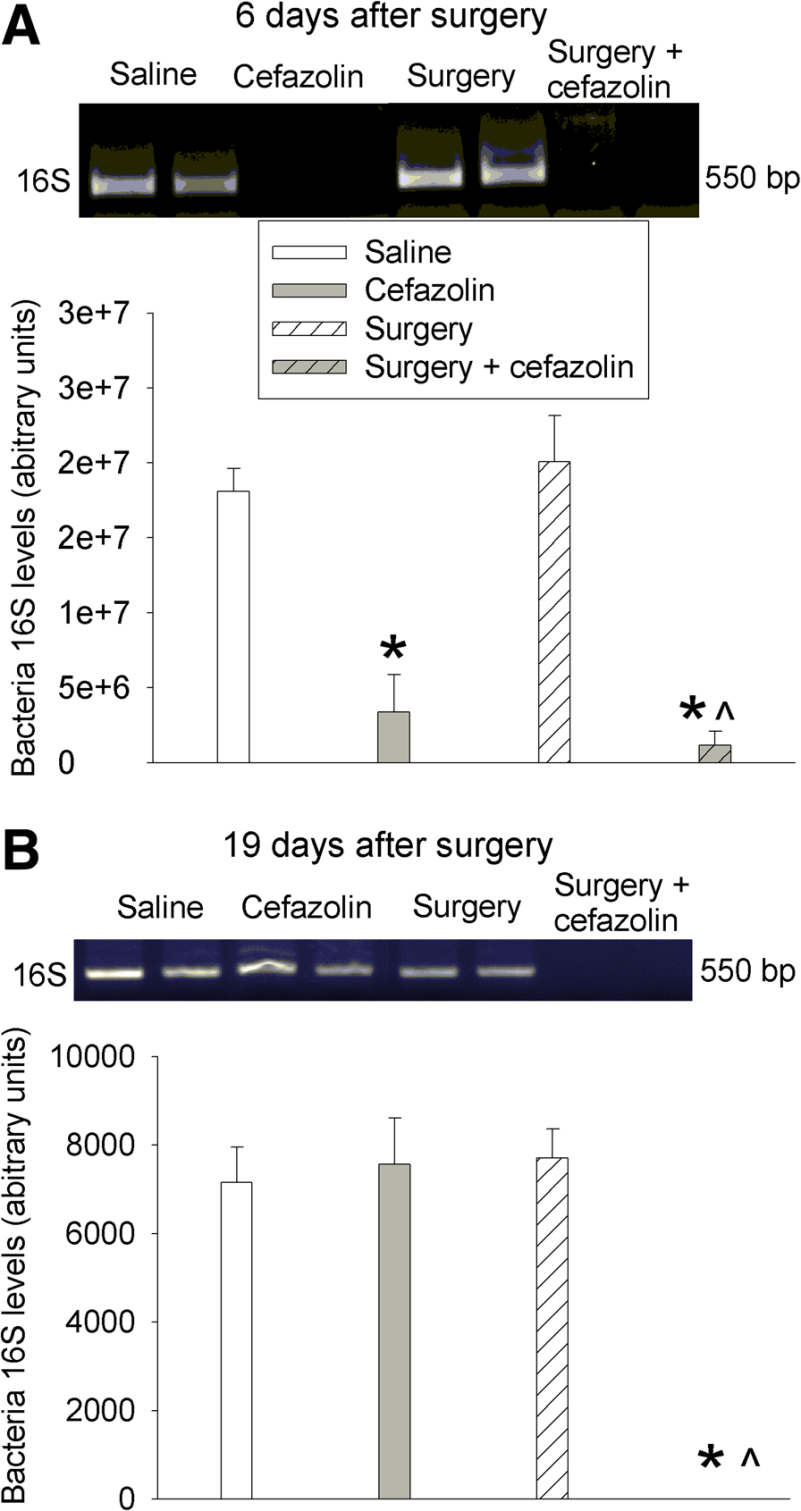
Effects of cefazolin and laparotomy on gut microbiota. Six- to 8-week-old CD-1 mice were subjected to laparotomy with or without cefazolin. The colon and its contents were harvested 6 days (**a**) or 19 days (**b**) after the surgery. The upper panel is a representative image of 16S rRNA PCR products. Results are mean ± S.E.M. (*n* = 5–7). *P < 0.05 compared with control mice, ^*P* < 0.05 compared with mice with laparotomy

Cefazolin decreased the expression of 16S rRNA that is expressed in almost all bacteria [26, 27]. This effect occurred no matter whether mice had surgery or did not have surgery. Surgery alone did not affect the expression of 16S rRNA (Fig. 6a). These results suggest that cefazolin induces gut dysbiosis. However, the expression of 16S rRNA in the mice with cefazolin alone recovered but was not recovered in mice with surgery and cefazolin at 19 days after the onset of cefazolin use (Fig. 6b). These results suggest that surgery delays the recovery of gut microbiota disrupted by cefazolin.

## Discussion

Minocycline, an antibiotic, has been shown to have a direct anti-inflammatory effect [15]. Our study showed that cefazolin reduced LPS-induced IL-6 and IL-1β production from C8-B4 mouse microglial cells. In addition, cefazolin attenuated surgery-induced increase of IL-6 in the cerebral cortex and IL-1β in the serum. These results suggest that cefazolin has a direct anti-inflammatory effect, which may be helpful to reduce tissue injury after surgery.

Surgery on peripheral tissues and organs induces systemic inflammation that can be transferred into the brain to induce neuroinflammation [28–30]. We and others have shown a critical role of neuroinflammation in POCD [5, 16, 17]. Since cefazolin has an anti-inflammatory effect, it is possible that cefazolin can reduce POCD. Consistent with this possibility, cefazolin attenuated surgery-induced learning and memory dysfunction. In addition, cefazolin attenuated surgery-induced increase of proinflammatory cytokines in the blood and brain 24 h after the surgery. Since cefazolin concentrations that have anti-inflammatory effects were achieved in the blood but were not reached in the brain after intraperitoneal injection of cefazolin and cefazolin application inhibited surgery-induced increase of proinflammatory cytokines in the blood, cefazolin may inhibit the systemic inflammatory response, which leads to the inhibition of neuroinflammation and improvement of learning and memory of mice after surgery. Cefazolin may not work directly on the brain to inhibit its inflammation.

To our surprise, we found that cefazolin impaired the freezing behavior of mice without surgery in the context-related fear conditioning test, suggesting a detrimental effect of cefazolin on learning and memory. Consistent with this suggestion, IL-1β was increased by cefazolin in the cerebral cortex 6 days after the surgery. More importantly, there was an increase in IL-6 and IL-1β in the colon of mice treated with cefazolin only on this day. Intraperitoneal injection of cefazolin almost eliminated the expression of 16S rRNA in the colon, suggesting a severe decrease of gut microbiota (dysbiosis). These results indicate the possibility that cefazolin induces gut dysbiosis that causes inflammatory responses in the gut and brain, which then induces learning and memory dysfunction.

Contribution of microbiota to brain health and diseases has been shown recently [12, 13]. Microbiota plays a critical role in normal brain development [31–33]. Changes in microbiota with aging may contribute to brain aging including learning and memory decline [13, 32], and cognitive functions are associated with the composition of the gut microbiota in elderly population [34]. Difference in microbiota has been found between healthy people and patients with neurodegenerative diseases, depression, and autism [13, 32, 35–37]. Mice which received sulfate-reducing bacteria have impaired working memory [38]. Transplanting microbiota from mice fed high-fat diet induces neuroinflammation and impairment of learning and memory in the recipient mice that do not have obesity [39]. These results indicate an important role of gut microbiome in learning, memory, and brain health. A recent study showed that depletion of gut microbiota with the combination of ampicillin, bacitracin, meropenem, neomycin, and vancomycin for 11 days impairs the novel object recognition memory [14]. Although the implication of this study in clinical setting is unknown because this combination of antibiotics is unlikely to be used in patients, the findings suggest that acute change of gut microbiota alters memory. Our findings indicate that systemic use of cefazolin, a commonly used method to reduce surgical infection, also induces acute changes in gut microbiota, inflammatory responses in gut and brain, and dysfunction of learning and memory.

Our results indicate that surgery alone induces gut inflammatory response because IL-6 and IL-1β in the colon were increased in mice with surgery alone. These mice may not have significant gut dysbiosis because the expression of 16S rRNA in these mice was similar to control mice. Thus, the inflammatory response in the gut of these mice with surgery may not be due to gut dysbiosis. It is unclear whether this inflammatory response in the gut is a local response to laparotomy or part of systemic response to this surgical procedure. Nevertheless, the acute phase of this inflammation in the gut was inhibited by cefazolin, consistent with the idea that this acute inflammatory response in the gut after surgery is not due to gut dysbiosis. Interestingly, the gut dysbiosis caused by cefazolin disappeared in mice treated with cefazolin alone but persisted in mice with surgery and cefazolin when the assay was performed 2 weeks after cefazolin application was stopped. These results suggest that surgery prolongs the recovery of gut microbiota.

We designed this study to simulate clinical setting. Intravenous injection of cefazolin is commonly used to prevent surgical infection [40, 41]. Although it may be used for a short period of time in relatively simple surgery with clear wound, its use for several days is not uncommon in patients with complex surgery or contaminated wound. Laparotomy, a procedure that is performed in all ages of patients, was used. This procedure is often performed in surgery involving organs in the abdominal cavity.

Our findings have significant implications. In addition to its effects on bacteria, cefazolin may have direct anti-inflammatory effect. This effect may explain its attenuation on anesthesia and surgery-induced learning and memory dysfunction. These effects may be beneficial to our patients. Our study also suggests that even routine systemic use of single antibiotic disturbs gut microbiota, which may lead to gut inflammatory response and cognitive dysfunction. Thus, caution must be taken when systemic or oral use of antibiotics is decided. Of note, it appears that the anti-inflammatory effect of cefazolin appears to dominate early in its use, but inflammatory response occurs when gut dysbiosis occurs at a delayed phase. Nevertheless, the gut dysbiosis appears to be transient when cefazolin is used alone.

Our study has limitations. First, we have not determined details of the changes in gut microbiota caused by cefazolin to implicate which bacteria may be involved in the effects of cefazolin on gut inflammatory responses, learning, and memory. The major goals of this initial study were to show the effects. Future studies will be performed to identify the bacteria involved. Second, our study used male mice only. Application of our findings to females needs to be cautious. However, it is hard to believe that cefazolin will not induce gut dysbiosis in female animals. Third, we used young adult mice but the delayed phase of POCD often occurs in elderly patients. However, acute phase of POCD at hospital discharge does occur in young patients [2]. Lastly, prolonged use of anesthetics can induce learning and memory dysfunction [42, 43]. We did not study the effects of mice with general anesthesia alone because we wanted to simulate clinical situation where general anesthesia is often used with surgery. In addition, patients with general anesthesia and without surgical incision may not need antibiotics.

## Conclusions

We have shown that cefazolin has a direct anti-inflammatory effect that may contribute to its attenuation of surgery-induced learning and memory dysfunction. However, systemic use of cefazolin can induce transient gut dysbiosis that may lead to neuroinflammation and the dysfunction of learning and memory. These novel findings indicate that perioperative antibiotic use may be beneficial from its anti-inflammatory effects but may cause transient gut dysbiosis when used alone.

## Abbreviations

IL-1β: Interleukin-1β
IL-6: Interleukin-6
LPS: Lipopolysaccharide
NS: Normal saline
POCD: Postoperative cognitive dysfunction
